# Evaluation of respiratory tract bacterial co-infections in SARS-CoV-2 patients with mild or asymptomatic infection in Lagos, Nigeria

**DOI:** 10.1186/s42269-022-00811-2

**Published:** 2022-04-21

**Authors:** Olabisi Flora Davies-Bolorunduro, Muinah Adenike Fowora, Olufemi Samuel Amoo, Esther Adeniji, Kazeem Adewale Osuolale, Oluwatobi Oladele, Tochukwu Ifeanyi Onuigbo, Josephine Chioma Obi, Joy Oraegbu, Oluwatobi Ogundepo, Rahaman Ademolu Ahmed, Olagoke AbdulRazaq Usman, Bosede Ganiyat Iyapo, Adedamola Adejuwon Dada, Ngozi Onyia, Richard Adebayo Adegbola, Rosemary Ajuma Audu, Babatunde Lawal Salako

**Affiliations:** 1 Microbiology Department, Nigerian Institute of Medical Research, Lagos, Nigeria; 2 Molecular Biology and Biotechnology Department, Nigerian Institute of Medical Research, Lagos, Nigeria; 3 Monitoring and Evaluation Unit, Nigerian Institute of Medical Research, Lagos, Nigeria; 4 Paelon Memorial Hospital, Lagos, Nigeria; 5 Federal Medical Centre, Ebute-Metta, Lagos, Nigeria

**Keywords:** Co-infection, Bacterial pathogens, SARS-CoV-2, COVID-19, Severity

## Abstract

**Background:**

A common complication of any respiratory disease by a virus could be a secondary bacterial infection, which is known to cause an increase in severity. It is, however, not clear whether the presence of some opportunistic pathogens called pathobionts contributes to the severity of the disease. In COVID-19 patients, undetected bacterial co-infections may be associated with the severity of the disease. Therefore, we investigated the implications of bacterial co-infections in COVID-19 cases.

**Results:**

This is a cross-sectional study that involved archived specimens collected from nasopharyngeal samples of 150 people for COVID-19 screening in Lagos. DNA extraction from the samples was carried out to determine the presence of five respiratory bacterial pathogens using nested real-time PCR, and data were analysed using the Chi-square test. Of the 150 samples collected, 121 (80.7%) were positive for SARs-CoV-2 infection and 29 were negative. The proportion of patients with bacteria co-infection in COVID-19-negative, asymptomatic, and mild cases were 93.1%, 70.7%, and 67.5%, respectively. There was no statistically significant difference between mild COVID-19 conditions and bacteria co-infection (*p* = 0.097). There was also no significant difference in the nasal carriage of *Staphylococcus aureus*, *Mycoplasma pneumoniae*, and *Haemophilus* spp. However, there was a statistically significant increase in the carriage of *Moraxella catarrhalis* and *Chlamydophila pneumoniae* among COVID-19-negative patients when compared with the positive patients (*p* value = 0.003 and 0.000 for *Moraxella catarrhalis* and *Chlamydophila pneumoniae*, respectively).

**Conclusions:**

The current study shows that bacterial co-infection and superinfection with COVID-19 are not associated with mild and asymptomatic COVID-19 cases in our setting. However, given the high prevalence of *Staphylococcus aureus* and *Mycoplasma pneumoniae* among the mild COVID-19 cases seen in this study, early diagnosis and treatment of these bacterial co-infections are still encouraged to mitigate the effect on the severity of COVID-19.

## Background

The coronavirus disease 2019 (COVID-19) which is caused by severe acute respiratory syndrome coronavirus 2 (SARS-CoV-2) is highly infectious. The disease emerged in Wuhan, China, and has become a pandemic (Shereen et al. 2020). Studies have shown that SARS-CoV-2and severe acute respiratory syndrome-like viruses (found in bats) are phylogenetically linked, and hence, bats could be the principal reservoir for SARS COV2 (Banerjee et al. 2019; Boni et al. 2020; Shereen et al. 2020). Epidemiologically, COVID-19 is currently a major pandemic with over 5.8 million deaths reported as of 15 February 2022 globally, which is of great concern (WHO Report 2022).

Secondary bacterial infections have been reported to be the cause of respiratory viral disease complications leading to increased morbidity in humans (Smith and McCullers 2014; Morris et al. 2017; Manohar et al. 2020). It was recently proposed that respiratory infections are linked to an imbalance of the nasopharyngeal microbiota (Man et al. 2017; Qin et al. 2020). Furthermore, increased bacterial colonization, immune dysfunction, and viral infection of the respiratory tract expose it to secondary bacterial infections (Morris et al. 2017). However, new data reveal that the microbial communities that live on our mucosal surfaces influence the rigor of our immune responses as well as the ecological connections that exist between hosts and infections (Chiu et al. 2017; Tay et al. 2020). In the last decade, there has been a lot of interest in figuring out how the microbial populations that live in our bodies regulate the balance between health and disease vulnerability, including infections. This suggests the notion that an acute viral infection disrupting normal microbial communities contributes to the development of post-viral bacterial pneumonia (Hanada et al. 2018).

Safaeyan et al. (2015) reported the presence of specific bacteria using qPCR technique in adult patients infected with influenza A, as well as healthy individuals. *Staphylococcus aureus, Streptococcus pneumoniae*, and *Haemophilus influenzae* were present in 12%, 24%, and 32% of infected patients, respectively, as compared to 5%, 11%, and 10% of uninfected patients.

It has been reported that pneumonia in older populations and young adults was associated with dysbiosis of the upper respiratory tract microbiome with bacterial overgrowth of a single species (de Steenhuijsen Piters et al. 2016).

It is, however, not clear whether the presence of some opportunistic pathogens called pathobionts contributes to the severity of the disease. Therefore, the study aimed at investigating and identifying bacterial pathogens or pathobionts in nasopharyngeal samples through direct amplification of their 16SrDNA genes from patients presenting symptoms of the COVID-19 could provide information regarding the probable relationship.

## Methods

### Ethics, study type, and study population

This study was submitted to the Institutional Review Board (IRB) of the Nigerian Institute of Medical Research (NIMR) and ethical approval was obtained from the (IRB/20/032) and written consent was obtained from the patients prior to sample collection.

This study was a cross-sectional study. A total of 150 nasopharyngeal samples obtained from participants who presented for COVID-19 testing during the period of May 2020 and August 2020 were retrieved from the NIMR biobank and included in the study.

### Sample collection

This step was carried out once for each patient. Briefly, nasopharyngeal samples were obtained using a swab and then placed into Viral Transport Medium (VTM) and taken to a Biosafety level III laboratory within the Institute. Information on demographic characteristics and symptoms presented was recorded during the patient’s enrolment.

### Reverse transcription real-time polymerase chain reaction (RT-PCR) for the detection of COVID-19

First, the extraction of the viral RNA was performed from the inactivated samples aliquots using QIAamp RNA extraction kit according to the manufacturer’s instructions (Qiagen, Maryland, USA). Next, one-step reverse transcriptase (RT) real-time (qPCR) was carried out to detect SARS-CoV-2 using qPCR assay designed by BGI, Shenzhen, China (Shaibu et al. 2021).

### Molecular analysis for bacterial identification

The method of Curran et al. (2007) was adopted with slight modification for the molecular analysis of the bacteria. Bacterial DNA was extracted from the sample, and species-specific primers of 5 bacteria known to be associated with respiratory tract infections (*S. aureus, M. catarrhalis, Haemophilus* sp., *C. pneumoniae*, and *M. pneumoniae*) were used for the amplification of the bacterial DNA using PCR (Table 1). The first reaction of the nested PCR was performed using the conventional PCR method in a Peltier PTC-200, MJ Research Thermal Cycler at an initial denaturation of 95 °C for 3 min, 35 cycles of 95 °C for 30 s, 55 °C for 40 s, 72 °C for 45 s, final elongation at 72 °C for 10 min, and a final hold at 10 °C for 0 s. The final reaction volume for the first round of the nested PCR was 20 µl which contained 4 µl of extracted DNA. The primers used for the target genes nuc, uspA, p6, momp, and p1 are listed in Table 1. The target genes were amplified using conventional PCR and nested real-time PCR. Master Mix (EvaGreen Solis Biodyne) made of 1 mM of each dNTP, 1X PCR buffer, 7.5 mM of Mgcl 2, and 1U of thermostable Taq polymerase, and 1 µM of each primer set (forward and reverse). Molecular grade water was used to make up the reaction mixture.

**Table 1:** Respiratory tract bacterial nested primers

S/N	Bacteria	Primer sequences (5’ → 3’)	Target
1	*Staphylococcus aureus*	F1-GCGATTGATGGTGATACGGTT R2-AGCCAAGCCTTGACGAACTAAAGC F2-TATGGCCCTGAAGCAAGTG R2-CGTTTACCATTTTTCCATCAGCA	Nuc
2	*Moraxella catarrhalis*	F1-CTGCCCAAGCTGCCCTAAGT R1-CAAAAGCCACAAAAACCC F2-GTGCTGGCTATCGTGTGAATCC R2-AAAAAGCAGCCAGTAAAC	uspA
3	*Haemophilus *spp*.*	F1-AACTTTTGGCGGTTACTCTG R1-CTAACACTGCACGACGGTTT F2-TTGGCGGATACTCTGTTGCTG R2-GTGCGCATCTAAGATTTGAACG	p6
4	*Chlamydophila pneumoniae*	F1-TTACAAGCCTTGCCTGTAGG R1-GCGATCCCAAATGTTTAAGGC F2-TTATTAATTGATGGTACAATA R2-ATCTACGGCAGTAGTATAGTT	Momp
5	*Mycoplasma pneumoniae*	F1-ATTCTCATCCTCACCGCCACCG R1-TGGTTTGTTGACTGCCACTGCCG F2-CAATGCCATCAACCCGCGCTTAACC R2-GTTGTCGCGCACTAAGGCCCACG	p1

All primers adapted from Curran et al. (2007)

The second reaction of the nested PCR was done using a BioRad CFX 96 Deep Well Real-Time system with HOT Firepol Evagreen ® qPCR supermix, 5× (cat no. 08-36-00008) according to the manufacturer’s instructions to identify the genetic expression in ct values of the genes specific to the bacteria using the primers. The supermix is a ready-to-use mix containing chemically modified Firepol® DNA Polymerase that enables hot start, optimized hot-start PCR buffer, Mgcl2, dUTPs, dNTPs, and Evagreen dye. The reaction was performed in a 20 µl reaction volume containing 0.1 µM Evagreen qPCR supermix (5×), 1 µM of each set of primers (forward and reverse), and 4 µl of DNA template from the first PCR reaction. The amplification was done at an initial denaturation of 95 °C for 5 min, followed by 30 cycles of 95 °C for 30 s, 55 °C for 30 s, and 72 °C for 30 s. The final elongation was done at 72 °C for 5 min. The temperature was raised to 95 °C for 30 s, 65 °C for 30 s, and the melt curve was performed at 0.5 °C increments of 65–95 °C every 1 s before the cycle was terminated.

### Quantitative nested real-time PCR

The method of Curran et al. (2007) was adopted with slight modification. Nested primers were designed targeting specific functional genes of a range of bacteria associated with respiratory infection (Table 1). The first round of each assay was carried out on a conventional thermal cycler (Peltier Thermal Cycler 200, MJ Research), while the second round of real-time PCR was carried out on an ABI 7000 system (Applied Biosystems, Warrington, UK). Each run included a negative control containing nuclease-free water as template. The 10-mL of PCR mastermix in the first-round contained two microlitres of either standard or sample DNA added to each reaction. The hot-start PCR cycling conditions were as follows: initial denaturation at 94 °C for 3 min; followed by 30 cycles of 94 °C for 30 s, 55 °C for 30 s, and 72 °C for 30 s; with a final extension step at 72 °C for 5 min.

The second-round real-time PCR mastermix was as above with 1_ SYBR Green I (Sigma). The template for the second round was 0.2 mL of the first-round reaction. The hot-start PCR cycling conditions were as follows: initial denaturation at 95 °C for 5 min; followed by 30 cycles of 95 °C for 30 s, 55 °C for 30 s, and 72 °C for 30 s; with a final extension step at 72 °C for 5 min. For melt-curve analysis, samples were denatured at 95 °C for 30 s, cooled at 65 °C for 30 s, and then slowly heated to 95 °C with continuous monitoring of fluorescence to determine the melting temperature of the DNA product. The results of the qPCR were analysed with the BioRad CFX Manager 3.1 software using the negative control to set the ct value.

### Statistical analysis

Demographic data such as age and gender were retrieved from records, de-identified, and presented in frequencies and percentages. Chi-square test was used to determine the association between the participants’ gender and age group of the severity of COVID-19 cases. The distribution and prevalence of bacterial co-infections with COVID-19 cases were evaluated using Chi-square test. Multivariate analysis (binary logistic regression) was used to ascertain the effects of age and gender on the likelihood of bacterial infection associated with COVID-19 severity. Also, SPSS software (version 23.0) was applied for all statistical analyses. Significant statistical associations were considered at a *p* value less than or equal to 0.05.

## Results

The prevalence of COVID-19 was higher in males than females. There is no statistically significant difference between the variables, gender, and severity of COVID-19 cases as *p* > 0.05. This implies that both males and females are equally likely to have severe cases of COVID-19. The association between participants’ age group and severity of COVID-19 cases is statistically significant as *p* ≤ 0.05, and this suggests that the variables, age group, and severity of COVID-19 cases are associated with each other. All the participants within the different age groups tend to exhibit dissimilar severe cases of COVID-19 (Table 2). The table depicts the severity level of cases such as mild, no symptoms as well as negative samples for the participant’s age group and gender.

**Table 2:** Association between gender, age group, and COVID-19 cases

Variables	COVID-19			*p* value
	Mild *n* (%)	No symptoms *n* (%)	Negative samples *n* (%)	
Gender				
Male	53(35.3)	25(16.7)	16(10.7)	0.553
Female	27(18.0)	16(10.7)	13(8.7)	
Age group				
< 20	1(0.7)	3(2.0)	0(0.0)	0.013^*^
20–40	37(24.7)	30(20.0)	19(12.7)	
41–61	37(24.7)	7(4.7)	9(6.0)	
> 61	5(3.3)	1(0.7)	1(0.7)	

^*^significant at 5% level

Generally, among study participants, the results showed that bacterial co-infection was present in 55.3% of the 150 samples with *Chlamydophila pneumoniae* being the most common bacterial co-infection among COVID-19 patients. Furthermore, there was a statistically significant increase in the carriage of *Moraxella catarrhalis* and *Chlamydophila pneumoniae* among COVID-19-negative patients when compared with the positive patients (*p* value was 0.003 and 0.000 for *Moraxella catarrhalis* and *Chlamydophila pneumoniae*, respectively) (Table 3). Of the 150 samples, 121 were positive for SARs-CoV-2 infection and 29 were negative. The COVID-19-negative patients had the highest prevalence of bacterial co-infections (93.1%) followed by asymptomatic COVID-19 patients (70.7%) and those presented with mild symptoms (67.5%). There was no statistically significant difference between COVID-19 severity and bacteria co-infection/superinfection (*p* = 0.097). The proportion of patient samples with at least one bacterial infection was found to be highest among those who presented with mild COVID-19 symptoms. There was a reduction in the proportion with a corresponding increase in the number of bacterial co-infections. The patients with mild symptoms were found to present the highest bacterial co-infections (1.2%) (Table 4). However, the patients with the highest prevalence of bacterial co-infections were those who tested negative at 93.1%. Overall, there was no association between the bacterial co-infections and COVID-19 cases.

**Table 3:** Distribution of bacteria among mild, asymptomatic, and negative COVID-19 patients

Nasal carriage of bacteria	COVID-19 Status			*p* value
	Positive (%)		Negative (%)	
	Mild symptoms	No symptoms		
*Moraxella catarrhalis*	33.3	27.3	39.4	0.003*
*Chlamydophila pneumoniae*	29.1	36.4	34.5	0.000*
*Staphylococcus aureus*	60.8	25.5	13.7	0.340
*Mycoplasma pneumoniae*	58.6	24.1	3.3	0.817
*Haemophilus *spp*.*	66.7	11.1	22.2	0.527

*Significant at 5% level

**Table 4:** Prevalence of bacterial co-infections in COVID-19 patients

COVID-19 status	Number of bacterial co-infections (%)						Proportion samples with bacterial infection (%)	*p* value
	0	1	2	3	4	5		
Mild (*n* = 80)	32.5	42.5	18.8	5.0	1.2	0	67.5	0.097
No symptoms (*n* = 41)	29.3	31.7	26.8	12.2	0	0	70.7	
Negative (*n* = 29)	6.9	41.4	41.4	10.3	0	0	93.1	

*Significant at 5% level

Regression models:

*Staphylococcus aureus* Infection = 1.243 − 0.216age − 0.015gender − 0.217severity

*Moraxella catarrhalis* Infection = 0.578 + 0.088age + 0.923gender + 0.499severity

*Chlamydophila pneumoniae* Infection = − 0.334 + 0.035age + 0.507gender + 1.314severity

*Mycoplasma pneumoniae* Infection = 2.792 − 0.310age − 0.788gender − 0.101severity

*Haemophilus *spp*.* Infection = 3.135 + 0.176age + 0.321gender − 1.270severity

The logistic regression model is a good fit since the log-likelihood value of the models is high with the model for *Staphylococcus aureus* having the highest value. Similarly, the Chi-square test showed that the model fits well as *p* > 0.05. The model for *Chlamydophila pneumoniae* explained 13.3% (Nagelkerke *R*^2^) of the variance in bacterial infections and correctly classified 75.2% of bacterial infections (Table 5). For the *Chlamydophila pneumoniae* model, males were 1.7 times more likely to get infected with *Chlamydophila pneumoniae* than females (OR = 1.66, 95% C.I. 0.698–3.950, *p* > 0.05). In addition, patients with mild COVID-19 cases were 3.7 times more likely to get infected with *Chlamydophila pneumoniae* than asymptomatic patients (OR = 3.721, 95% C.I. 1.551–8.926, *p* < 0.05). Increasing age and gender was associated with an increased likelihood of COVID-19 patients getting infected with *Chlamydophila pneumoniae*. Similarly, increasing severity of COVID-19 cases was associated with an increased likelihood of COVID-19 patients getting infected with *Chlamydophila pneumoniae* (Table 6).

**Table 5:** Fitted binary logistic regression of bacterial infection associated with COVID-19 severity

Model	Predictors	− 2*LL*(*θ*)	${\chi }^{2}$	PA (%)	${R}_{N}^{2}$
*Staphylococcus aureus*	Age, gender, severity of COVID-19 cases	157.540	4.265^a^	63.6	0.012
*Moraxella catarrhalis*	Age, gender, severity of COVID-19 cases	103.562	0.628^a^	83.5	0.068
*Mycoplasma pneumoniae*	Age, gender, severity of COVID-19 cases	116.656	1.912^a^	80.2	0.050
*Haemophilus *spp*.*	Age, gender, severity of COVID-19 cases	51.730	2.946^a^	94.2	0.040

− 2*LL*(*θ*): log likelihood of the model; ${\upchi }^{2}$: Chi-square test statistic value; PA[%]: percentage accuracy in classification; ${R}_{N}^{2}$: Nagelkerke determination coefficient

^a^*p* > 0.05, OR (odds ratio)

**Table 6:** Logistic estimates for the binary logistic regression of bacterial infection associated with COVID-19

	Characteristics of patients	${\beta }_{i}$	${SE}_{{\beta }_{i}}$	Wald statistic (df)	*p* value	Odd ratio	95% C.I for OR	
							Lower	Upper
*Staphylococcus aureus*	Constant	1.243	0.757					
	Age	− 0.216	0.321	0.454(1)	0.500	0.806	0.430	1.511
	Gender	− 0.015	0.412	0.001(1)	0.971	0.985	0.439	2.209
	Severity	− 0.217	0.428	0.256(1)	0.613	0.805	0.348	1.864
*Moraxella catarrhalis*	Constant	0.578	0.964					
	Age	0.088	0.435	0.041(1)	0.840	1.091	0.466	2.559
	Gender	0.923	0.517	3.182(1)	0.074	2.517	0.913	6.940
	Severity	0.499	0.534	0.873(1)	0.350	1.647	0.578	4.687
*Chlamydophila pneumoniae*	Constant	− 0.334	0.819					
	Age	0.035	0.362	0.009(1)	0.924	1.035	0.509	2.105
	Gender	0.507	0.442	1.313(1)	0.252	1.660	0.698	3.950
	Severity	1.314	0.446	8.664(1)	0.003^*^	3.721	1.551	8.926
*Mycoplasma pneumoniae*	Constant	2.792	0.969					
	Age	− 0.310	0.384	0.649(1)	0.420	0.734	0.346	1.558
	Gender	− 0.788	0.558	1.996(1)	0.158	0.455	0.152	1.357
	Severity	− 0.101	0.530	0.036(1)	0.849	0.904	0.320	2.556
*Haemophilus *spp*.*	Constant	3.135	1.688					
	Age	0.176	0.675	0.068(1)	0.794	1.192	0.317	4.479
	Gender	0.321	0.825	0.152(1)	0.697	1.379	0.274	6.949
	Severity	− 1.270	1.132	1.258(1)	0.262	0.281	0.031	2.584

${\beta }_{i}$: estimated coefficients, ${SE}_{{\beta }_{i}}$: standard error of the estimated coefficients

*Significant at 5% level

## Discussion

In this study, we investigated the presence of bacterial co-infections in COVID-19 cases and analysed their epidemiological characteristics. There have been several reports of prevalence of COVID-19 to be higher in males than females (Jin et al. 2020; Abate et al. 2020; Huang et al. 2021). Data from this study revealed the prevalence of COVID-19 to be higher in males than females. This agrees with the findings of Sharifipour et al. (2020) where 58% of the COVID-19-positive patients were male and 42% were female. This could be attributed to the fact that females present a higher immune response than males (Takahashi et al. 2020). Also, gender-based lifestyles such as higher levels of smoking among men compared to women and women having a more responsible attitude towards the COVID-19 pandemic than men in their undertaking of preventive measures such as wearing face masks and frequent handwashing may be contributing factors (Bwire 2020).

Findings from this study revealed that age group 20–61 had the highest prevalence of COVID-19 particularly among those who presented with mild symptoms. This is not surprising as the working-class group falls within this age category and they could have been predisposed to the virus through commuting to work in comparison with the younger age group as well as the elderly who were mostly at home during the partial lockdown period at the time.

Bacterial co-infections have been linked to viral disease severity, both directly and indirectly as well as via immunological response (DaPalma et al. 2010; Alosaimi et al. 2020; Mirzaei et al. 2020). Several studies have reported the prevalence of influenza/bacterial co-infection as well as multiple viral co-infections (Alosaimi et al. 2020; D’Abramo et al. 2020; Ding et al. 2020; Konala et al. 2020). However, data on clinical significance of bacterial co-infections with COVID-19 are limited and the few available studies detected the bacterial pathogens through culture-based methods. Our study investigated the coexistence of 5 bacteria respiratory pathogens and COVID-19 simultaneously in order to understand the relationship between bacterial infection and a possible increase in severity of the disease. Previous studies have revealed that secondary bacterial co-infection is the main cause of death in patients with viral pneumonia (MacIntyre et al. 2018; Guo et al. 2020). In this study, bacterial co-infection was present in 55.3% of the study participants and the most common bacterial co-infection among COVID-19 patients was *Chlamydophila pneumoniae*.

*Moraxella catarrhalis* was observed in the study to be significantly more in SARS-CoV-2-negative cases. Other reports show prevalence of *Moraxella catarrhalis* to be higher in COVID-19-positive cases with less than 5% (Massey et al. 2020; Feldman and Anderson 2021). Our result is, however, in agreement with Calcagno et al. (2021) where *Moraxella catarrhalis* was higher in COVID-19 cases. The ubiquitous surface protein A2 (UspA2) on *Moraxella catarrhalis* is very important for its bactericidal activity as well as cross-reactive antibodies. It is usually a negative regulator of TLR3, and IL-1β consequently could reduce the antiviral defence function of the epithelium and cause an increase in viral infections susceptibility (Heinrich et al. 2016; Ysebaert et al. 2021).

Though *Staphylococcus aureus*, *Mycoplasma pneumoniae* and *Haemophilus* spp recorded the highest prevalence among the group which presented with mild COVID-19 symptoms, there was also no statistically significant difference in the nasal carriage of these bacteria among cases with both asymptomatic and mild symptoms. Our findings appeared to be in concordance with previous findings from Saudi Arabia but inconsistent with findings from China (Xing et al. 2020) where *Mycoplasma pneumoniae* and *Legionella pneumophila* were the most common bacteria detected among COVID-19 patients. This could be attributed to the diversity of geographical distribution of circulating respiratory bacteria. Nevertheless, in this study, *Chlamydophila pneumoniae* infection has been associated with mild COVID-19 symptoms. This, however, is a cause for concern and the bacterium has been reported as a common cause of acute respiratory infections with a seroprevalence of 34.1% in COVID-19 patients with fatalities (Du et al. 2020).

Our study has some limitations. First, only 5 bacteria were identified. Second, our study did not include severe and critical cases thereby limiting the highest severity level to patients with mild symptoms. Future studies to overcome these limitations need to be considered.

## Conclusions

Our study highlights the importance of screening for co-infecting bacteria in COVID-19 patients, given the high prevalence of *Chlamydophila pneumoniae* infection. As there is a continuous increase in antimicrobial resistance, the detection of bacterial co-infections in COVID-19 cases shows the importance of early diagnosis and commencement of early treatment with antibiotics to reduce possible impact on the severity of the disease by hospitalization and associated mortality. Further studies of the respiratory tract microbiome in relation to respiratory illnesses (COVID-19) using a modern molecular approach such as next-generation sequencing (NGS) which would identify other pathobionts of the respiratory tract are recommended.

## Data Availability

The datasets used and/or analysed during the current study are included in the article.
